# A prognostic 15-gene model based on differentially expressed genes among metabolic subtypes in diffuse large B-cell lymphoma

**DOI:** 10.3389/pore.2023.1610819

**Published:** 2023-02-02

**Authors:** Jun Hou, Peng Guo, Yujiao Lu, Xiaokang Jin, Ke Liang, Na Zhao, Shunxu Xue, Chengmin Zhou, Guoqiang Wang, Xin Zhu, Huangming Hong, Yungchang Chen, Huafei Lu, Wenxian Wang, Chunwei Xu, Yusheng Han, Shangli Cai, Yang Liu

**Affiliations:** ^1^ Department of Pathology, Sichuan Cancer Hospital and Institute, Sichuan Cancer Center, School of Medicine, University of Electronic Science and Technology of China, Chengdu, China; ^2^ Burning Rock Biotech, Guangzhou, China; ^3^ Medical Oncology, Sichuan Cancer Hospital and Institute, Sichuan Cancer Center, School of Medicine, University of Electronic Science and Technology of China, Chengdu, China; ^4^ Department of Clinical Trial, The Cancer Hospital of the University of Chinese Academy of Sciences (Zhejiang Cancer Hospital), Hangzhou, China; ^5^ Institute of Basic Medicine and Cancer (IBMC), Chinese Academy of Sciences, Hangzhou, China

**Keywords:** prognosis, risk score, drug sensitivity, DEGs, diffuse large B-cell lymphoma (DLBCL), metabolic subtypes

## Abstract

The outcomes of patients with diffuse large B-cell lymphoma (DLBCL) vary widely, and about 40% of them could not be cured by the standard first-line treatment, R-CHOP, which could be due to the high heterogeneity of DLBCL. Here, we aim to construct a prognostic model based on the genetic signature of metabolic heterogeneity of DLBCL to explore therapeutic strategies for DLBCL patients. Clinical and transcriptomic data of one training and four validation cohorts of DLBCL were obtained from the GEO database. Metabolic subtypes were identified by PAM clustering of 1,916 metabolic genes in the 7 major metabolic pathways in the training cohort. DEGs among the metabolic clusters were then analyzed. In total, 108 prognosis-related DEGs were identified. Through univariable Cox and LASSO regression analyses, 15 DEGs were used to construct a risk score model. The overall survival (OS) and progression-free survival (PFS) of patients with high risk were significantly worse than those with low risk (OS: HR 2.86, 95%CI 2.04–4.01, *p* < 0.001; PFS: HR 2.42, 95% CI 1.77–3.31, *p* < 0.001). This model was also associated with OS in the four independent validation datasets (GSE10846: HR 1.65, *p* = 0.002; GSE53786: HR 2.05, *p* = 0.02; GSE87371: HR 1.85, *p* = 0.027; GSE23051: HR 6.16, *p* = 0.007) and PFS in the two validation datasets (GSE87371: HR 1.67, *p* = 0.033; GSE23051: HR 2.74, *p* = 0.049). Multivariable Cox analysis showed that in all datasets, the risk model could predict OS independent of clinical prognosis factors (*p* < 0.05). Compared with the high-risk group, patients in the low-risk group predictively respond to R-CHOP (*p* = 0.0042), PI3K inhibitor (*p* < 0.05), and proteasome inhibitor (*p* < 0.05). Therefore, in this study, we developed a signature model of 15 DEGs among 3 metabolic subtypes, which could predict survival and drug sensitivity in DLBCL patients.

## Introduction

Diffuse large B-cell lymphoma (DLBCL), a type of highly heterogeneous cancer, accounts for 30%–40% of non-Hodgkin lymphoma (1). The prognosis of DLBCL varies due to its distinct characteristics such as clinical factors, recurring mutations, cell of origin (COO) (2), etc. Currently, while approximately 60% of DLBCL patients could be cured by R-CHOP (a combined therapy of rituximab, cyclophosphamide, doxorubicin, vincristine, and prednisone), the rest still suffer from a poor prognosis with fatal recurrent or progressive disease (3). Hence, effective prognostic stratification systems for DLBCL could benefit these patients in clinical decision-making and treatment.

At present, there are several commonly used prognosis-related classification systems for DLBCL in clinical practice, especially International Prognostic Index (IPI) (4) and COO (5, 6). Although IPI is convenient for application, its limitations are also obverse, in which only clinical factors are used and heterogeneous features of DLBCL, such as intrinsic genes and other biomarkers, are not considered. In the COO classification system, DLBCL is divided into germinal center B-cell-like (GCB) and non-GCB including activated B-cell-like (ABC) and type-III DLBCL based on immunohistochemistry algorithms (5) or gene expression profiling (GEP) analysis (6). Patients with GCB-DLBCL have better outcomes than those with non-GCB (6). However, the COO classification is mainly based on the B cell receptor (BCR) signaling pathway (7), and the isotype of BCR alone can predict the prognosis similar to COO (8). In addition, neither IPI nor COO classification can predict the efficacy of most drugs, although BTK inhibitor (BTKi)' ibrutinib, was reported more effective in ABC than GCB DLBCL in a phase 1/2 clinical trial (9). Therefore, prognostic models of novel biomarkers are needed to assist the therapeutic drug selection in DLBCL.

Cancer cells autonomously alter their metabolic flux to meet the demands for rapid growth and survival, including increased bioenergetic and biosynthetic, mitigating oxidative stress, and immune evasion, etc. (10). For instance, the Warburg effect is a classic alteration in carbohydrate metabolism in tumors (11). Conversely, aberrantly accumulated metabolites also promote tumorigenesis (12). Targeting metabolic alterations has been considered a promising therapeutic strategy in some cancer types (13, 14). Meanwhile, there is a great interest in exploiting the relationship between metabolic gene expression and cancer prognosis stratification both in pan-cancer (15, 16, 17) and single solid tumors (18, 19, 20). In DLBCL, metabolism heterogeneity was reported a long time ago (21). However, just limited attention has been paid to correlating risk signatures to metabolic alterations. Therefore, the heterogeneity of metabolic expression profiles could be a novel perspective on prognosis stratification for DLBCL patients.

In this study, we identified three subtypes of DLBCL based on expression levels of genes in metabolism pathways. With the selected DEGs among these subtypes, we developed a risk score model to help stratify the survival of DLBCL patients and analyzed the relationships between the risk score and clinicopathological characteristics, drug sensitivity, and immune cell infiltrations. Our research provided a new prognosis model for DLBCL patients, rendering novel insights into the individual management of DLBCL.

## Methods

### Data obtaining

Microarray gene expression profiles and corresponding clinical-related information of the training dataset (GSE31312) and four validation datasets (GSE10846, GSE53786, GSE87371, and GSE23501) were downloaded from the NCBI Gene Expression Omnibus (GEO) database (https://www.ncbi.nlm.nih.gov/geo/). Excluding 0-month survival samples, 470, 412, 119, 221, and 69 DLBCL patients were respectively included in GSE31312, GSE10846, GSE53786, GSE87371, and GSE23501. For genes with multiple probes, the median value of gene expression was used in the following analyses. The clinicopathological data of the patients in each dataset were summarized in Supplementary Table S1.

### Metabolic subtype classification

Overall, 1,916 metabolic genes of 7 major metabolic processes from Peng et al. (16) were initially studied and listed in Supplementary Table S2. The expression profiles of these 1,916 metabolic genes in GSE31312 were employed to perform Partitioning Around Medoids (PAM) clustering by the “ConsensusClusterPlus (v1.50.0)” R package with Euclidean distance. The distribution of clinical characteristics of patients was analyzed among subtypes. The “survival” and “survminer” R packages were used to analyze survival differences among subtypes, and the result was shown by the Kaplan-Meier survival curve.

### ssGSEA

Single sample gene set enrichment analysis (ssGSEA) was applied by the “GSVA” R package using the 50 hallmark gene sets from the Molecular Signatures Database (MSigDB, http://www.gsea-msigdb.org/gsea/index.jsp). The result was shown as a heatmap by the “ComplexHeatmap” R package.

### Differentially expressed gene (DEG) analysis

Fold change (FC) was calculated by dividing the mean value of gene expression in one subtype group by that in the other subtype group. Wilcoxon signed-rank test was employed to calculate *p* value. The genes with |log_2_FC| > 0.585 and Benjamini-Hochberg-adjusted *p* < 0.05 were identified as DEGs. For DEGs in these subtype groups, KEGG and GO enrichment analyses were performed independently using “clusterProfiler” R package.

### Construction and validation of the risk score model

First, the *p* value of <0.01 was used as the threshold to select DEGs with prognostic significance in univariable Cox regression analysis. Then, the selected DEGs were analyzed by least absolute shrinkage and selection operator (LASSO)-Cox regression analysis through the “glmnet” and “survival” packages. Ten-fold cross-validation was employed to determine the penalty parameter (λ) of the prognostic model and followed minimum criteria. The formula below was to calculate the risk score based on the expression level of each gene and its corresponding regression coefficient:

$$Risk\;Score=\overset{n}{\underset{i=0}{\sum }}\beta_{i}*\chi_{i}$$

*β*_*i*_: weight coefficient of each gene; *χ*_*i*_: expression quantity of each gene.

The high- and low-risk groups were divided according to the median value. Overall survival (OS) and progression-free survival (PFS) of patients between the two groups were compared by the Kaplan-Meier survival curves using the “survminer” package. Then, the signature was validated in four external datasets (GSE10846, GSE53786, GSE87371, and GSE23051).

Univariable and multivariable Cox analyses were conducted to determine whether the risk score was an independent prediction factor of OS and PFS in the training and four validation cohorts. Meanwhile, receiver operating characteristic (ROC) curves in the above datasets were constructed and determined the AUC values through the “timeROC” package.

### Association analysis between clinicopathological characteristics and risk score

The risk scores were compared in different groups of age, sex, stage, extranodal sites, ECOG score, IPI, and GEP subtype, separately.

### Drug sensitivity analysis

The drug sensitivity of different risk groups was predicted by the data from The Genomics of Drug Sensitivity in Cancer (GDSC) database (https://www.cancerrxgene.org/) (22). The half maximal inhibitory concentration (IC50) was analyzed using “pRRophic” R package (23).

### Immune cell infiltration analysis

According to the LM22 gene signature of tumor-infiltrating immune cells (TIICs) pattern for distinguishing human immune cell phenotypes (24), the fraction of TIICs was analyzed by CIBERSORT (the Cell-type Identification by Estimating Relative Subsets of RNA Transcripts, http://cibersort.stanford.edu/).

### Statistical analysis

Statistical analyses and mapping of data were performed by the R software (version 4.1.2; https://www.R-project.org). Continuous variables were compared by Mann-Whitney U test or Kruskal-Wallis test. Categorical variables were compared by Fisher’s exact test. A two-tailed *p* value <0.05 indicated statistical significance.

## Results

### Classification of metabolic subtypes and their prognostic differences

**FIGURE 1:**
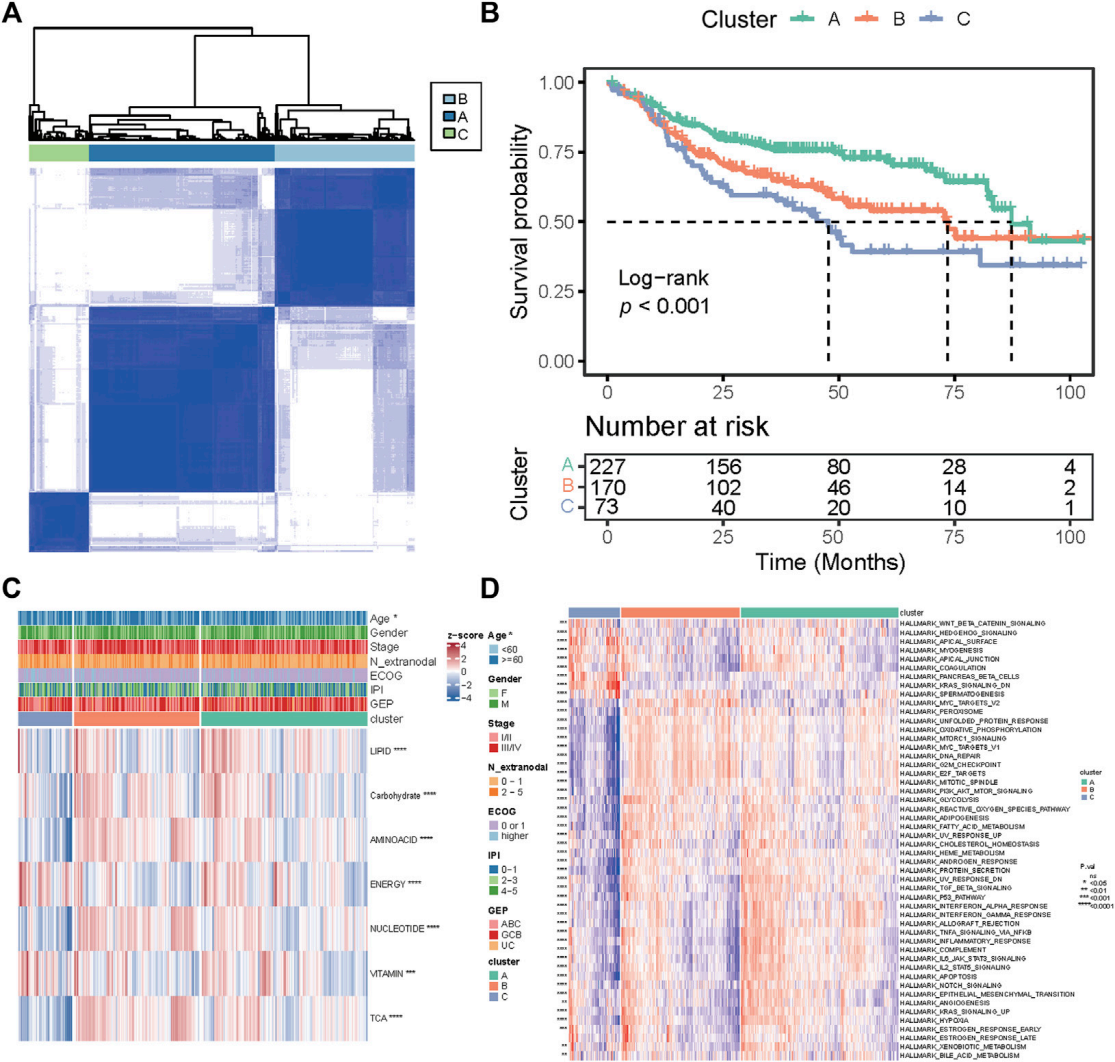
Classification of metabolic subtypes and their prognostic difference in the GSE31312 dataset. **(A)** Consensus matrix heatmap of three metabolic expression clusters. **(B)** Kaplan–Meier curves of OS among the three clusters. **(C)** Heatmap of seven metabolic pathway expression patterns of the three clusters. *p < 0.05; **p < 0.01; ***p < 0.001; ****p < 0.0001. **(D)** Heatmap of expression patterns of 50 hallmark gene sets of the three clusters. *p* value was measured by the log-rank test.

The overall study plan was depicted in Supplementary Figure S1. To characterize the metabolic heterogeneity of DLBCL patients, we analyzed 1,916 genes in 7 metabolic pathways (16) in the GSE31312 cohort, including 766 genes in the lipid metabolism pathway, 286 genes in the carbohydrate metabolism pathway, 348 genes in the amino acid metabolism pathway, 110 genes in the integration of energy pathway, 90 genes in the nucleotide metabolism pathway, 168 genes in the vitamin cofactor metabolism pathway, and 148 genes in the tricarboxylic acid cycle (TCA cycle) pathway. Using an unsupervised consensus algorithm, 470 patients were divided into 3 metabolic subtypes (clusters A, B, and C with 227, 170, and 73 patients, respectively) in the training cohort (Figure 1A). The respective median OS of clusters A, B, and C was 87.3 months, 73.5 months, and 47.7 months (Log-rank *p <* 0.001; Figure 1B). Notably, the median OS of cluster C was significantly shorter than those of clusters A and B.

We further investigated the differences in pathway enrichment among the three metabolic subtypes. We found that compared with clusters A and B, cluster C had lower expression of genes in the nucleotide metabolism, TCA cycle, and amino acid metabolism and higher expression of genes in the integration of energy and vitamin cofactor metabolism (Figure 1C). The clinical variable analyses showed that patients in cluster C were older (*p* = 0.044) than those in other clusters (Figure 1C). Then, we conducted ssGSEA to assess the differential expression levels of 50 biological hallmarks in the three groups. The expression pattern of those hallmarks in cluster C was different from that in other clusters (Figure 1D). These results indicate that the differences in the expression of genes in seven major metabolic pathways could stratify the prognosis of DLBCL.

### Identification of DEGs and functional annotation

**FIGURE 2:**
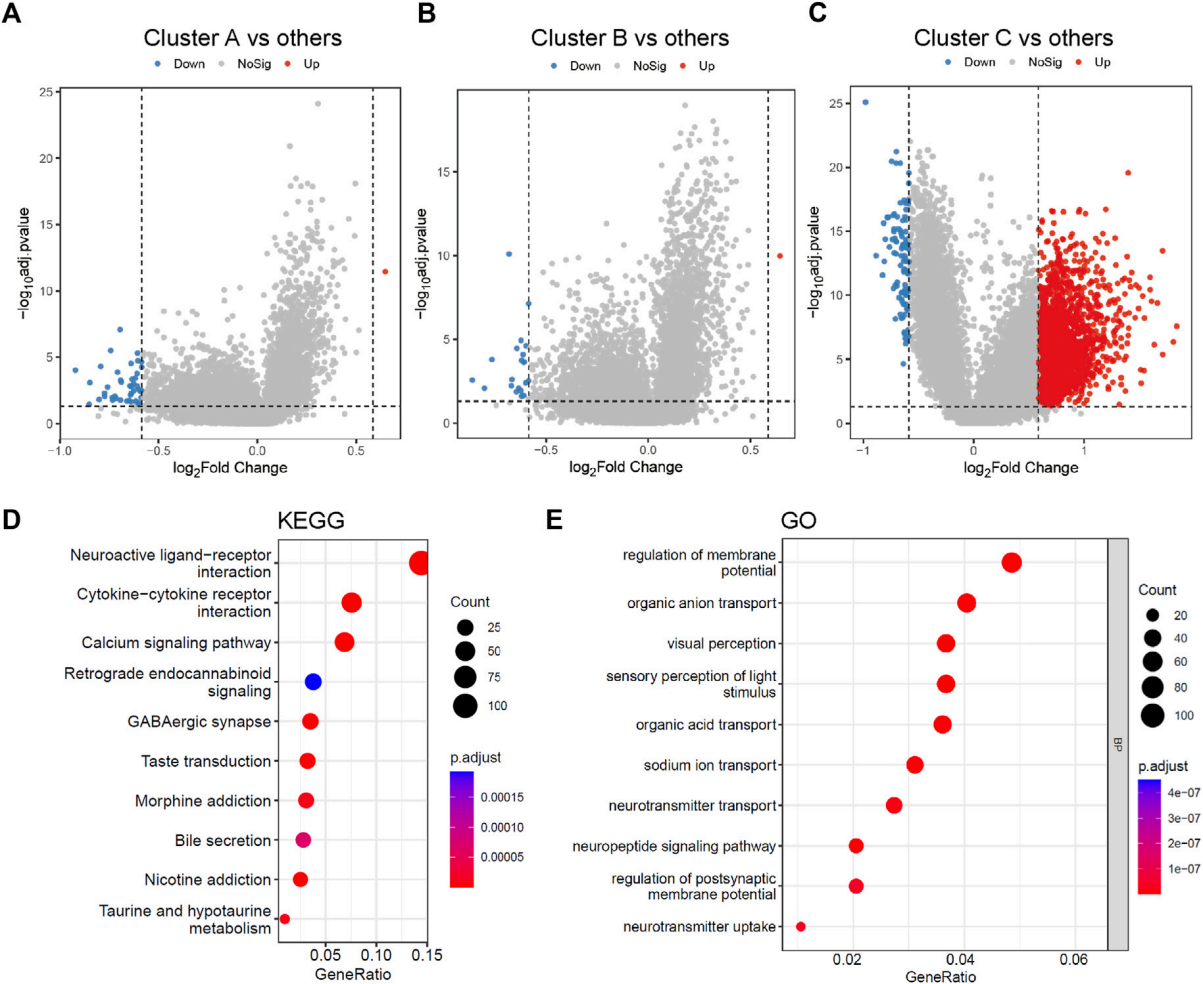
Differentially expressed genes among three metabolic subtypes and gene function enrichment analysis. **(A–C)** Volcano plots of the DEGs between cluster A and the other two clusters, cluster B and the other two clusters, cluster C and the other two clusters, separately. **(D,E)** KEGG and GO analysis of DEGs between cluster C and the other two clusters. The size of the bubbles denotes the number of genes enriched in the corresponding pathways, and the difference in color represents distinct significance. KEGG, Kyoto Encyclopedia of Genes and Genomes; GO, Gene Ontology; BP, biological process.

To select the metabolic genetic signature, DEG analysis was conducted and identified 1,854 DEGs. There were 43, 21, and 102 downregulated genes and 1, 1, 1,743 upregulated genes in clusters A, B, and C, respectively (Figures 2A–C, Supplementary Table S3). Functional analyses of those DEGs *via* KEGG pathway and GO analyses were not able to provide any results for clusters A and B, probably due to the small sizes of DEGs sets (44 vs. 22). DEGs in cluster C were more related to neuroactive ligand-receptor interaction, cytokine-cytokine receptor interaction, and calcium signaling pathway (Figure 2D). Biological processes associated with the regulation of membrane potential, organic anion transport, organic acid transport, and sodium ion transport were also enriched in cluster C (Figure 2E).

### Construction of the risk score model

**FIGURE 3:**
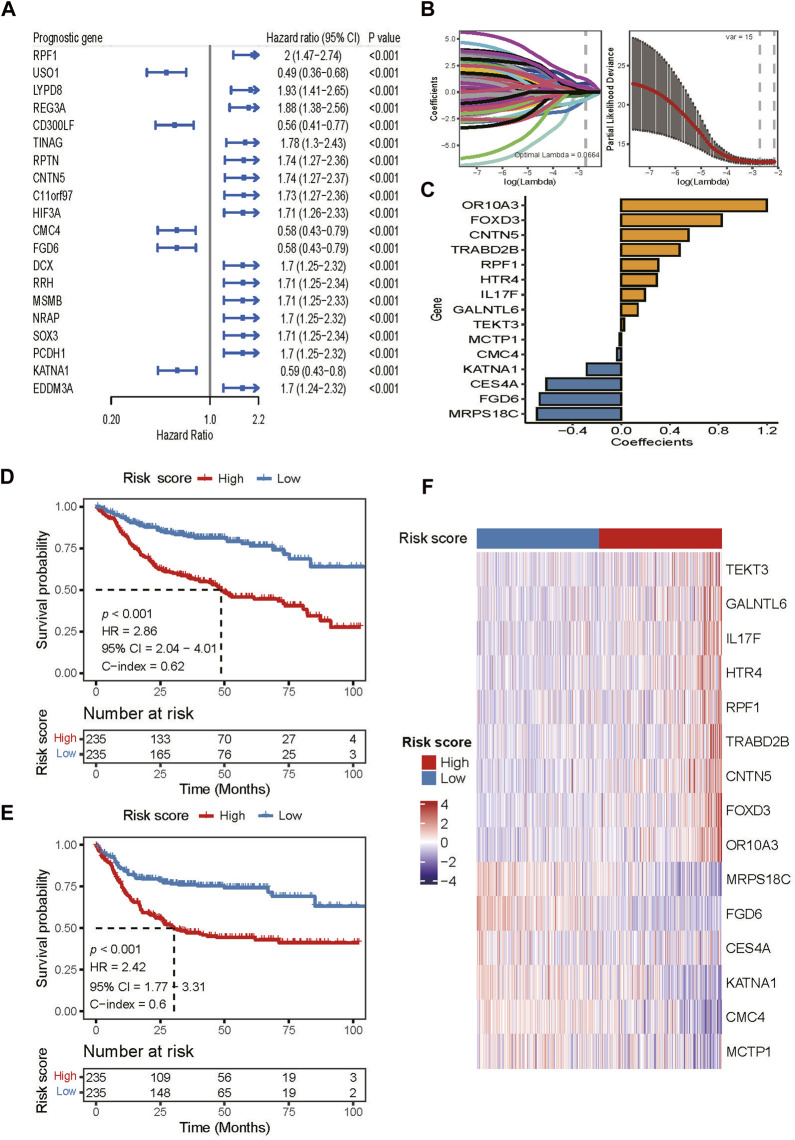
Construction of the risk score model in the training dataset. **(A)** Top 20 of the 108 prognosis-related DEGs identified by univariable Cox regression analysis. **(B)** LASSO regression analysis of the 108 prognosis-related DEGs. **(C)** Fifteen DEGs and their coefficients used for constructing the risk score. **(D,E)** Kaplan-Meier curves of OS and PFS of patients in the training dataset assigned to high and low-risk groups. **(F)** Heatmap of gene expression patterns of the 15 model-used genes in patients assigned to high- and low-risk groups.

To construct a risk score model, we analyzed 1,854 DEGs identified above using univariable Cox regression. The threshold of *p* < 0.01 were used to screen genes that were most related to the prognosis of DLBCL patients. A total of 108 prognosis-associated genes were selected in the training set (Supplementary Table S4). The top-20 prognosis-associated genes, according to the significance level (*p* value), are listed in Figure 3A. Then, we performed LASSO penalty regression to construct the prognostic model in the training set (Figure 3B). A subset of 15 genes and their weighting coefficients were finally identified (Figure 3C; Supplementary Table S5). Furthermore, the risk score of individual patients was calculated, and all patients were dichotomized into high- or low-risk groups according to the median value of the risk score.

In the training dataset, patients in the high-risk group had both shorter OS (HR 2.86, 95% CI 2.04–4.01; *p* < 0.001; Figure 3D) and PFS (HR 2.42, 95% CI 1.77–3.31; *p* < 0.001; Figure 3E) than those in the low-risk group. The areas under the receiver operating characteristic curve (AUCs) for 1-, 3-, and 5- years OS were 0.701, 0.703, and 0.724, respectively (Supplementary Figure S2A). For 1-, 3-, and 5- years PFS, the AUCs were 0.667, 0.699, and 0.685, respectively (Supplementary Figure S2B). Moreover, the gene expression heatmap revealed that the high expressions of genes, *OR10A3*, *FOXD3*, *CNTN5*, *TRABD2B*, *RPF1*, *HTR4*, *IL17F*, *GALNTL6*, and *TEKT3* were observed in the high-risk group, while in the low-risk group, *MCTP1*, *CMC4*, *KΑTNA1*, *CES4A*, *FGD6*, and *MRPS18C* were highly expressed (Figure 3F).

### Validation of risk score model in the independent validation cohorts

**FIGURE 4:**
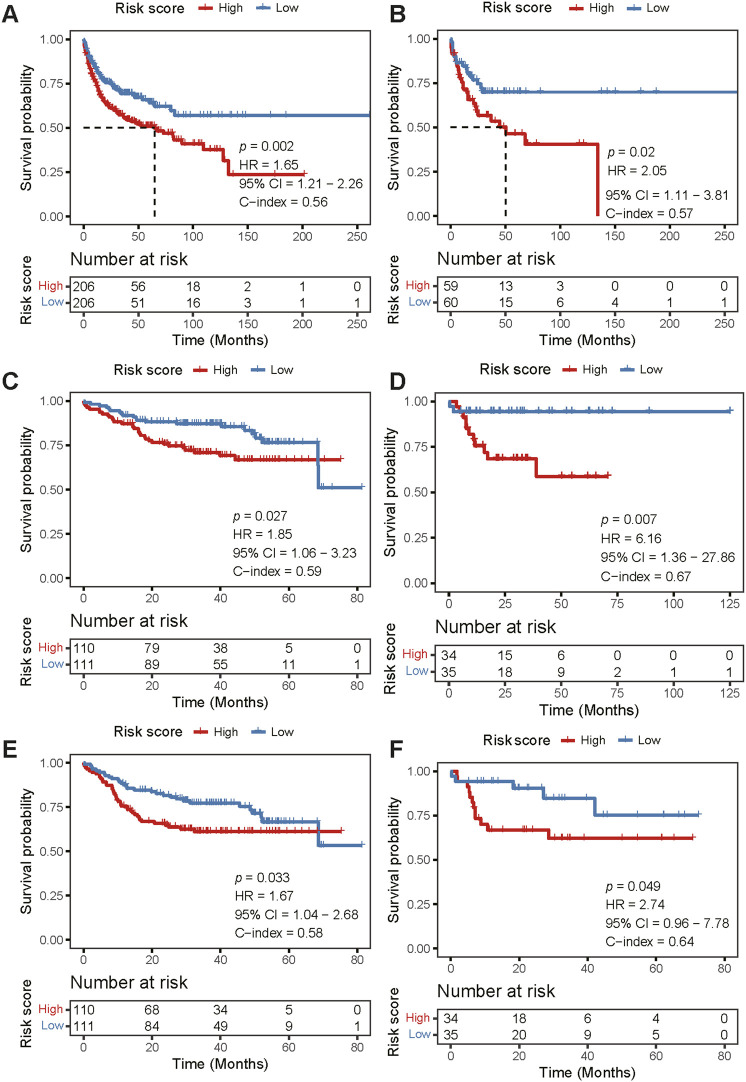
External validation of the risk score model. **(A–D)** Kaplan-Meier curve analysis of OS between the high- and low-risk groups in four independent validation cohorts [GSE10846 **(A)**, GSE53786 **(B)**, GSE87371 **(C)**, and GSE23501 **(D)**, respectively]. **(E,F)** Kaplan-Meier curve analysis of PFS between the high- and low-risk groups in two independent cohorts [GSE87371 **(E)** and GSE23501 **(F)**, respectively]. There was no PFS information in GSE10846 and GSE53786. OS, overall survival; PFS, progression-free survival.

We further validated the risk score model in four external cohorts, the robustness of the prognostic model was supported by significant prognostic values for OS in GSE10846 (HR 1.65, 95% CI 1.21–2.26; *p* = 0.002; Figure 4A), GSE53786 (HR 2.05, 95% CI 1.11–3.81; *p* = 0.02; Figure 4B), GSE87371 (HR 1.85, 95% CI 1.06–3.23; *p* = 0.027; Figure 4C), and GSE23051 (HR 6.16, 95% CI 1.36–27.86; *p* = 0.007; Figure 4D). For PFS validation, the results showed the same trend with statistical significance (GSE87371: HR 1.67, 95% CI 1.04–2.68; *p* = 0.033; Figure 4E; GSE23051: HR 2.74, 95% CI 0.96–7.78; *p* = 0.049; Figure 4F). In the validation datasets, the AUCs of the model were higher in GSE23501 for both OS (Supplementary Figures S2C–F) and PFS (Supplementary Figures S2G, H) for 1-, 3-, and 5- years. Therefore, compared with the high-risk group, patients in the low-risk group had better outcomes, indicating the predictive potential of our model.

### Independent prognostic role of the risk score model

The independent prognostic value of the model was further studied, taking into consideration of age, sex, stage, ECOG, and IPI. The risk score was verified to be an independent prognostic factor of OS in all cohorts in both univariable and multivariable Cox regression analyses (*p* < 0.05; Supplementary Figures S3A–E) and an independent prognostic factor of PFS in GSE31312 (*p* < 0.001; Supplementary Figure S3F) and GSE87371 (*p* = 0.015; Supplementary Figure S3G). As expected, IPI was also an independent factor to predict PFS and OS (Supplementary Figure S3).

### Association between clinicopathological characteristics and risk score

Further analysis of clinical characteristics showed that the risk score was higher in patients with the following characteristics: age >60 years (*p* = 0.0018), stage III/IV disease (*p* = 0.029), >1 extranodal sites (*p* = 0.024), higher IPI scores (*p* = 0.00017), or cluster C (*p* < 0.01) (Supplementary Figure S4).

### Association between drug sensitivity and risk score

**FIGURE 5:**
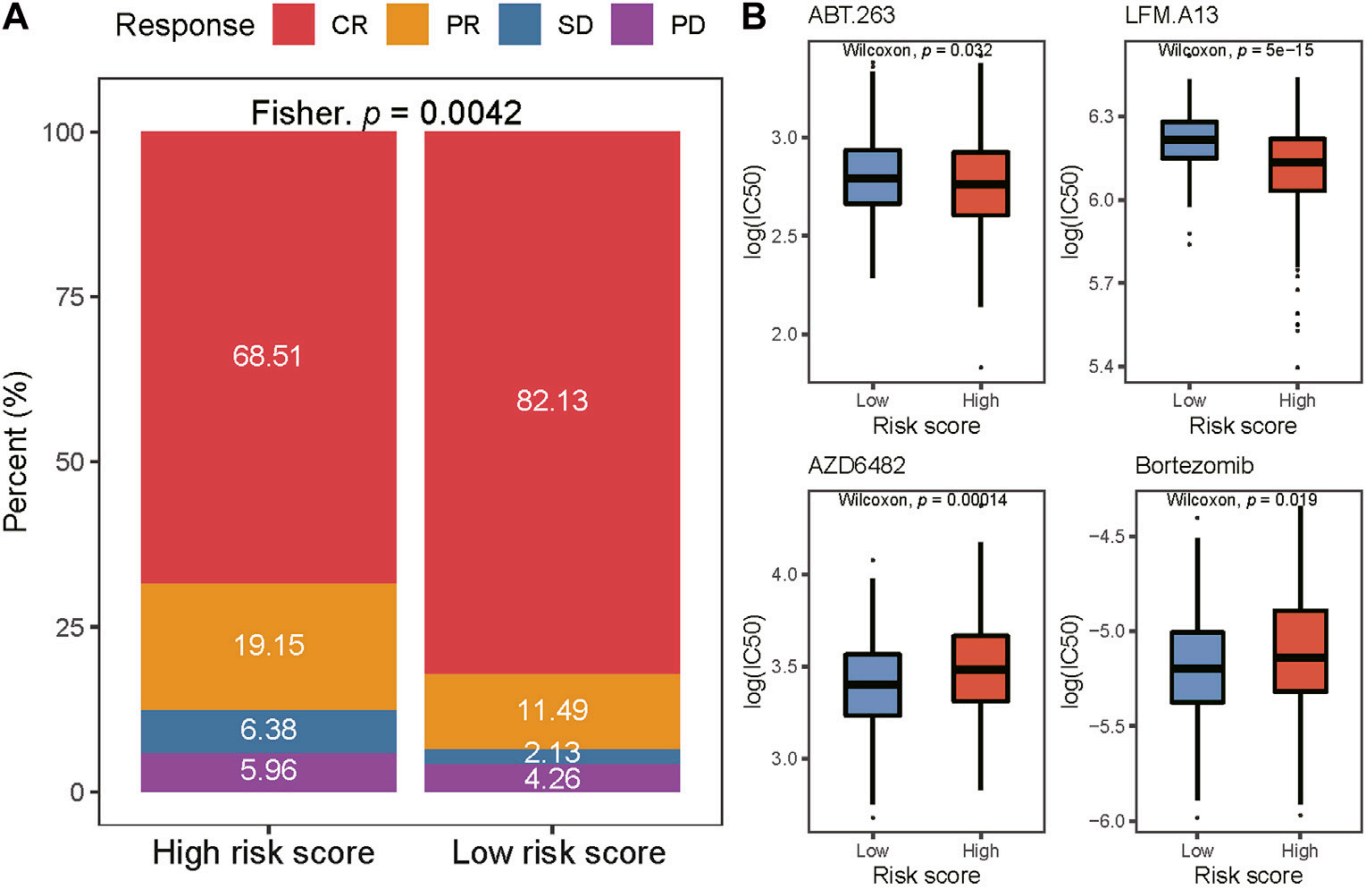
Comparison of drug sensitivity between two risk groups. **(A)** Distribution of CR, PR, SD, and PD in two risk groups. CR complete response; PR partial response; SD stable disease; PD progressive disease. **(B)** The IC50 of ABT263., LFM. A13, AZD6482, and Bortezomib in low-and high-risk groups. IC50, the half maximal-inhibitory concentration.

All patients in the training cohort were treated with the R-CHOP regimen, the first-line standard-of-care treatment for DLBCL (25). We studied the response rates to the R-CHOP in the high- and low-risk groups. We found that the complete response (CR) rate was higher in the low-risk group (82.1% vs. 68.5%, *p* = 0.0042; Figure 5A). Then, we used the data from the GDSC database to predict the response to targeted agents in the two risk groups (Figure 5B). The estimated IC50s for BCL2i (ABT.263) and BTKi (LFM.A13) were lower in the high-risk group (*p* < 0.05), while estimated IC50s for PI3K inhibitor (PI3Ki and AZD6482) and proteasome inhibitors (Bortezomib) were lower in the low-risk group (*p* < 0.05).

### Characteristics of immune cell infiltration of the two risk score groups

**FIGURE 6:**
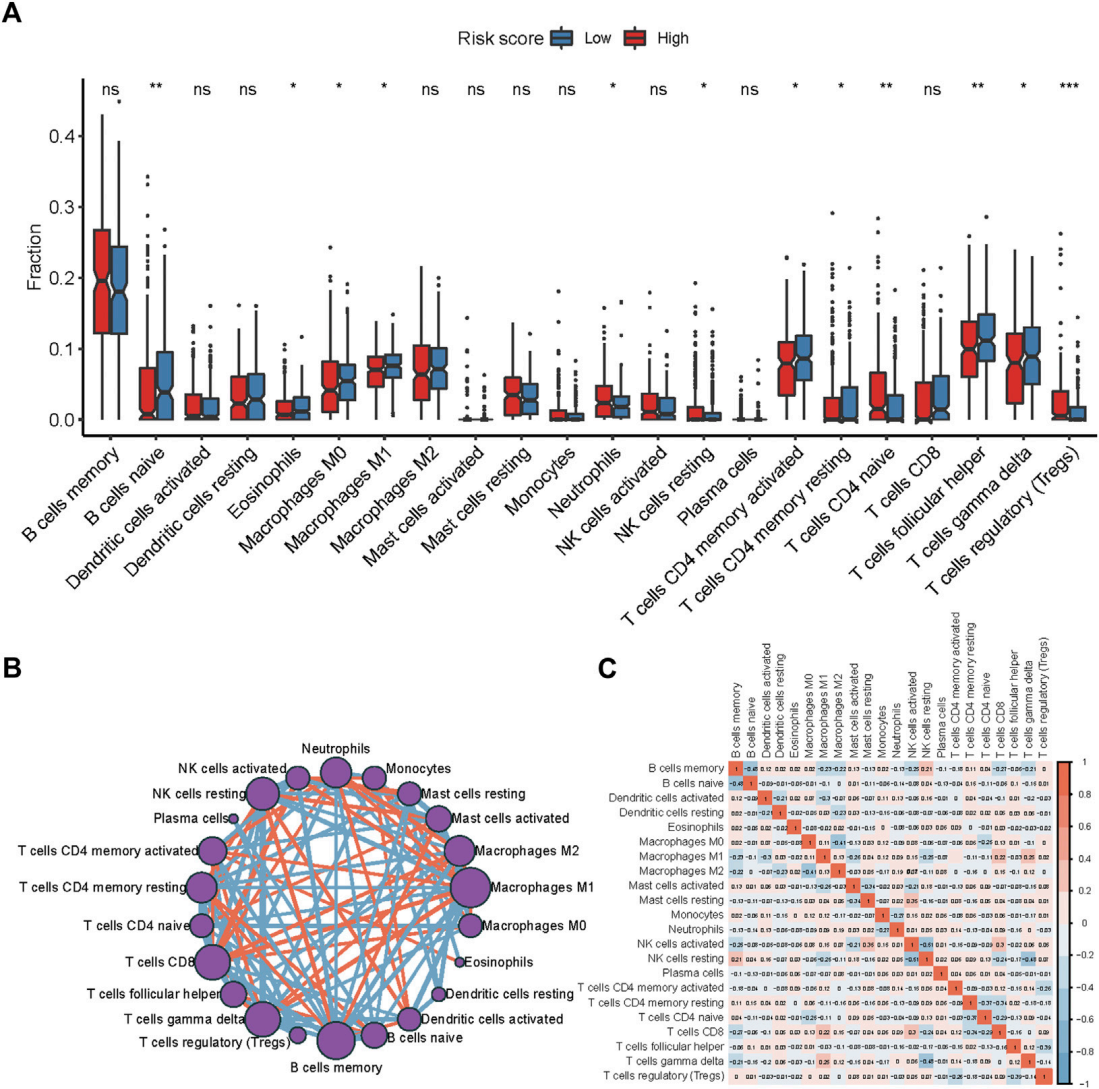
Immune cell infiltration discrepancy between two risk groups. **(A)** Comparison of the infiltration of 22 types of immune cells in the low- and high-risk groups. *p < 0.05; **p < 0.01; ***p < 0.001 by the Wilcoxon test. **(B)** Chord diagram of the correlation among 22 leukocyte subtypes in patients from the GSE31312 cohort. **(C)** The correlation diagram of the immune cells from the GSE31312 cohort.

Tumor-infiltrating immune cells (TIICs), a component of the tumor microenvironment (TME), have been found to be associated with prognosis and treatment response. To explore the relationship between the risk score and TIICs, we analyzed the discrepancy of immune cell infiltration between two risk groups according to the LM22 gene signature[24] (Figure 6A). The proportion of naïve B cells, eosinophils, M1 Macrophages, activated CD4 memory T cells, resting CD4 memory T cells, follicular helper T cells, M0 Macrophages, and gamma delta (γδ) T cells were significantly higher in the low-risk group (*p* < 0.05). By contrast, neutrophils, resting NK cells, naïve CD4 T cells, and regulatory T cells (T-regs) were notably higher in the high-risk group (*p* < 0.05). In the training cohort, M1 macrophages, memory B cells, γδ T cells, activated CD4 memory T cells, neutrophils, and CD8 T cells were found at the core of the correlation network (Figure 6B). The correlation heatmap showed that activated CD4 memory T cells correlated positively with γδ T cells and negatively with T-regs (Figure 6C, Supplementary Figure S5). Altogether, these results suggest a remarkable discrepancy in immune cell infiltration between the high- and low-risk groups while the potential mechanism may be complex.

## Discussion

DLBCL is a challenge in individualized treatments due to its significant heterogeneity. Although R-CHOP can cure over 60% of patients (2, 3) using traditional stratification systems like IPI (26) and COO (5, 6), a limitation of these systems is evident, either neglect biological factors (26) or only focus on the BCR signal (7, 8). Importantly, none of them can predict drug response. Metabolic signatures have been proposed for prognosis in many other neoplasms (18, 19, 27, 28) but not in DLBCL yet. Therefore, we distinguished three metabolic subtypes with different prognoses in DLBCL. Among them, 108 prognosis-associated DEGs were identified, of which 15 genes were used to construct a risk score model of DLBCL. The model showed a robust ability to predict the outcomes of DLBCL independently. As expected, there were distinctly different immune cell infiltration and clinicopathological characteristics between the high- and low-risk groups. The results also imply that patients in the high-risk group predictively respond to BCL2 and BTK inhibitors, while patients in the low-risk group might consider PI3Ki and proteasome inhibitors.

In this study, we explored the metabolic genetic features in DLBCL and identified three subtypes of patients with distinct survival. The most distinct differences were in cluster C, which had the shortest OS with lower expression of genes in nucleotide metabolism, TCA cycle, or amino acid metabolism and higher expression of genes in the integration of energy and vitamin cofactor metabolism. Interestingly, another metabolic expression subtype of 32 cancers also showed the clinical outcomes of subtypes with upregulated vitamin/cofactor metabolism were worse, while subtypes with upregulated nucleotide metabolism had a better prognosis (16). However, there were some inconsistent results. For instance, increased expressions of numerous nucleotide metabolism genes were associated with worse outcomes in breast cancer patients (29). The inconsistency may attribute to the following reasons: 1) previous studies merely focused on one or several metabolites and genes of an individual metabolic pathway (29,30,31), 2) signaling pathways may interact with each other (20, 32), and 3) the same signaling could play distinct roles in different diseases. In summary, the results suggest metabolic heterogeneity in DLBCL can be used for prognostic stratification though it needs more validation.

Based on DEGs among the three metabolic subtypes, a risk score model of 15 genes was developed. Among the 15 genes, *OR10A3*, *FOXD3*, *CNTN5*, *TRABD2B*, *RPF1*, *HTR4*, *IL17F*, *GALNTL6*, and *TEKT3* were highly expressed in the high-risk group, which was associated with poor prognosis, while the high expression of *MRPS18C*, *FGD6*, *CES4A*, *KΑTNA1*, *CMC4*, and *MCTP1* were characterized in the low-risk group and related to favorable prognosis. Among the poor prognosis-related genes, *TRABD2B* (also known as *TIKI2*), *IL17F*, and *GALNTL6* were reported to be related to the oncogenesis of renal cell carcinoma (33), cutaneous T-cell lymphoma (34), and thyroid carcinoma (35), respectively. Moreover, the mutations in *CNTN5* were reported to contribute to the metastatic process of pancreatic cancer (36), *HTR4* was found predominantly in only high-grade prostate cancer (37, 38), and the high expression of *TEKT3* could be influenced by HBV integration events in liver cancer (39). Therefore, these genes promoting pathogenesis or progression in several cancers may also lead to poor survival in DLBCL. However, *FOXD3,* a poor prognosis gene in our model, was reported as a suppressor factor of *H pylori* infection-induced gastric carcinoma (40) and melanoma (41). One possible explanation for this difference is the dual role of forkhead Box D3 encoded by *FOXD3*. Forkhead Box D3, a member of the forkhead family of transcription factors, can function as both a transcriptional repressor and activator. Of the favorable prognosis-related genes, previous studies have reported that the downregulated *MCTP1* was related to drug-resistance of esophageal cancer (42), and *MRPS18C* was the least expressed MRPS18 family member in malignantly transformed B-cells (43). Thus, the higher expression of these genes may be associated with favorable prognosis. The expression of *FGD* genes, a gene family comprising *FGD6*, were analyzed to predict the OS of head and neck squamous cell carcinoma (HNSC) (44), in which the OS was positively related to high expression of *FGD2* and *FGD3* but not *FGD6*. The favorable effect of *FGD6* on prognosis in DLBCL needs further investigation. In addition, the over-expression of *CMC4* (also known as *MTCP1*), as a favorable prognosis gene in our model, was discordantly reported to produce clonal CD5^+^/CD19^+^ leukemia in mice (45), which was thought to be a chronic lymphocytic leukemia driving gene. For the remaining genes, *OR10A3*, *RPF1*, *KΑTNA1*, and *CES4A*, in our risk score model, no specific relationship to cancer had been reported yet, further exploration should be carried out for their roles in the prognosis in DLBCL patients.

The relationship of risk score with clinical characteristics and treatment response was explored in our study. Patients with an age older than 60 years, advanced stage (stage III/IV), and high IPI scores had higher risk scores. Older than 60 years, LDH (lactate dehydrogenase) greater than normal, PS score of 2‒4 points, stage III/IV, and more than 1 extranodal sites are well-known high-risk factors in DLBCL (4), which is consistent with our results. Meanwhile, a greater proportion of patients in the cluster C subtype was observed in the high-risk group, which is also consistent with our result that cluster C was a poor prognosis factor in our research. In addition, our results showed that low-risk patients had a significantly higher CR rate than high-risk patients after R-CHOP treatment. Previous studies have confirmed that the higher CR/CRu rate of DLBCL patients after chemotherapy would improve the overall survival (46, 47), suggesting that the higher sensitivity to the R-CHOP regimen in the low-risk group may be another reason for its favorable prognosis.

DLBCL is a heterogeneous lymphoma (48). In this study, we observed immune cell infiltration discrepancy in two risk groups. A higher proportion of activated CD4 memory T cells, M1 macrophages, and γδ T cells were in the low-risk group relevant to better prognosis. These results were consistent with previous studies. Chen et al. reported that when patients had a higher proportion of CD4 memory T cells and γδ T cells, they were more sensitive to R-CHOP regimen so that more patients achieved CR/PR (49). Another study also showed that activated CD4 memory T cell was an independent factor of favorable prognosis in DLBCL patients (50). The reason may be that after chemotherapy, CD4^+^ T cells can produce multiple proinflammatory cytokines including IFNγ, TNFα, and IL2 (51). These factors may allow patients to achieve durable remissions through CD8^+^ effector cell-mediated antitumor immunity (51). For γδ T cells, their cytotoxic effect and ability to secrete IFN can generate antitumor effects (52). Its subgroup, Vγ9Vδ2T cells, can increase antibody dependent cellular cytotoxicity (ADCC) of rituximab and further enhance the efficacy of the R-CHOP regimen (53). Meanwhile, Yan et al. (54) found that M1 macrophage infiltration was related to a lower risk of progression and improved overall survival, as FCγR-dependent stimulation of M1 macrophage mediated ADCP (antibody-dependent cellular phagocytosis) maintained anti-lymphoma activity. In addition, T-regs were highly expressed in the high-risk group, suggesting that T-regs are relevant to poor prognosis. The prognostic role of T-regs is now a matter of debate. Autio et al. (55) found in the Nordic Lymphoma Group trial cohort (NCT01325194), a higher proportion of T-regs was associated with worse prognosis, but this could not be repeated in the Helsinki study cohort (NCT01502982). These controversial results may be caused by the heterogeneity of DLBCL. Interestingly, in this study, we found that CD4 memory-activated T cells were positively related to γδ T cells but negatively correlated with T-regs. This mechanism of the correlation between those immune cells is needed in the future.

In our study, we also explored the potential response of the high- and low-risk groups to targeted drugs, in which BCL2 inhibitor and PI3K inhibitor were suggested for the high- and low-risk groups, respectively. Interestingly, in the previous classification based on genetic heterogeneity (3), the BCL2 SVs were associated with poor outcomes of GCB-DLBCLs and PI3K with good-risk GCB-DLBCLs, while BCL2 was considered in the EZB subtype of DLBCL with favorable outcomes in another genetic classification (56). A therapeutic classification of DLBCL was constructed based on the responses to drugs targeted at genetic alteration (57) and both BCL2 inhibitor and PI3K inhibitor were suggested for the MCD subgroup with poor survival and EZB subgroup with good survival. Hopefully, combining the study of Chapuy et al. (3), our results would provide more clues to make the decision of treatment for DLBCL.

Some limitations are in our study. Although we included a total of 1,291 patients and 4 independent validation sets, which suggests that the results may be highly reliable, the risk score model should be further verified through a prospective study. Second, our study was based on bioinformatic analyses of public data, while validations by clinical specimens are needed to be studied. Last, the mechanism of how the 15 genes in the risk score model affect prognosis in DLBCL needs to be further explored.

## Conclusion

Overall, we identified three metabolic subtypes in DLBCL patients with different clinical outcomes and further constructed a prognostic 15-gene model based on DEGs among the three subtypes, which indicates that the differentially expressed gene profile of metabolic heterogeneity may provide a new strategy for prognosis stratification in DLBCL patients. Additionally, the risk score model demonstrated a remarkable predictive value of survival and drug sensitivity, which may benefit individualized prognosis management and personalized therapeutic intervention in DLBCL.

## Data Availability

The datasets generated and analyzed in the present study are available in the public data repository, GEO: https://www.ncbi.nlm.nih.gov/geo/.
