# Supplementary material for: A prognostic 15-gene model based on differentially expressed genes among metabolic subtypes in diffuse large B-cell lymphoma
Source: Pathol Oncol Res. 2023 Feb 2;29:1610819. doi: 10.3389/pore.2023.1610819 (PMC9931744; doi:10.3389/pore.2023.1610819)
Supplement: Supplementary file 3 [file Table1.DOCX]

Table S1. The clinicopathological data of patients in the training and validation cohorts.

| **Characteristics** | **Training dataset** | **Validation datasets** | | | |
| --- | --- | --- | --- | --- | --- |
|  | GSE31312  (n = 470) | GSE87371  (n = 221) | GSE53786  (n = 119) | GSE23501  (n = 69) | GSE10846  (n = 412) |
| **Age** |  |  |  |  |  |
| <60 | 186 (40%) | 109 (49%) | 53 (45%) | 26 (38%) | 179 (43%) |
| >=60 | 284 (60%) | 112 (51%) | 66 (55%) | 43 (62%) | 233 (57%) |
| **Gender** |  |  |  |  |  |
| Female | 199 (42%) | 105 (48%) | 48 (40%) | 19 (28%) | 172 (42%) |
| Male | 271 (58%) | 116 (52%) | 68 (57%) | 50 (72%) | 222 (54%) |
| Unknown |  |  | 3 (3%) |  | 18 (4%) |
| **Stage** |  |  |  |  |  |
| I/II | 220 (47%) | 71 (32%) |  |  | 188 (46%) |
| III/IV | 250 (53%) | 150 (68%) |  |  | 217 (53%) |
| Unknown |  |  |  |  | 7 (1.7%) |
| **Extranodal sites** | |  |  |  |  |
| 0 | 194 (41%) |  | 63 (53%) |  | 236 (57%) |
| 1 | 172 (37%) |  | 33 (28%) |  | 115 (28%) |
| 2 | 66 (14%) |  | 6 (5.0%) |  | 19 (4.6%) |
| 3 | 27 (5.7%) |  | 2 (1.7%) |  | 8 (1.9%) |
| 4 | 8 (1.7%) |  |  |  | 2 (0.5%) |
| 5 | 3 (0.6%) |  |  |  | 1 (0.2%) |
| N/A |  |  | 11 (9.2%) |  | 31 (7.5%) |
| Unknown |  |  | 4 (3.4%) |  |  |
| **IPI** |  |  |  |  |  |
| 0-1 | 169 (36%) | 74 (33%) |  | 12 (17%) |  |
| 2-3 | 197 (42%) | 98 (44%) |  | 40 (58%) |  |
| 4-5 | 58 (12%) | 49 (22%) |  | 13 (19%) |  |
| Unknown | 46 (10%) |  |  | 4 (5.8%) |  |
| **OS** |  |  |  |  |  |
| 0 | 300 (64%) | 168 (76%) | 75 (63%) | 56 (81%) | 249 (60%) |
| 1 | 170 (36%) | 53 (24%) | 44 (37%) | 13 (19%) | 163 (40%) |
| **PFS** |  |  |  |  |  |
| 0 | 292 (62%) | 150 (68%) |  | 52 (75%) |  |
| 1 | 178 (38%) | 71 (32%) |  | 17 (25%) |  |
| **OS time (Days)** | 1,051  (519, 1,699) | 1,083  (676, 1,479) | 701  (294, 1,455) | 686  (343, 1,194) | 859  (338, 1,564) |
| **PFS time (Days)** | 814  (327, 1,520) | 972  (444, 1,460) |  | 664  (270, 1,048) |  |
| **Response** |  |  |  |  |  |
| CR | 354 (75%) |  |  |  |  |
| PD | 24 (5.1%) |  |  |  |  |
| PR | 72 (15%) |  |  |  |  |
| SD | 20 (4.3%) |  |  |  |  |
| **COO (Hans algorithm)** | |  |  |  |  |
| GC | 248 (53%) |  |  |  |  |
| Non-GC | 222 (47%) |  |  |  |  |
| **COO** |  |  |  |  |  |
| ABC-like | 199 (42%) |  |  |  |  |
| GCB-like | 227 (48%) |  |  |  |  |
| Unclassified | 44 (9.4%) |  |  |  |  |
| **ECOG performance status** | | |  |  |  |
| 0 or 1 | 374 (80%) |  | 73 (61%) |  | 295 (72%) |
| higher | 96 (20%) |  | 35 (29%) |  | 93 (23%) |
| N/A |  |  | 8 (6.7%) |  | 24 (5.8%) |
| Unknown |  |  | 3 (2.5%) |  |  |
| n (%); Median (IQR). IQR, interquartile range. IPI, International Prognostic Index; OS, overall survival; PFS, progression-free survival; CR, complete remission; PD, progressive disease; PR, partial remission; SD, stable disease; COO, cell-of origin classification; GC, germinal center; ABC, activated B cell-like; GCB, germinal center B cell-like; ECOG, Eastern Cooperative Oncology Group. | | | | | |
