# Supplementary figures and images for: A prognostic 15-gene model based on differentially expressed genes among metabolic subtypes in diffuse large B-cell lymphoma
Source: Pathol Oncol Res. 2023 Feb 2;29:1610819. doi: 10.3389/pore.2023.1610819 (PMC9931744; doi:10.3389/pore.2023.1610819)

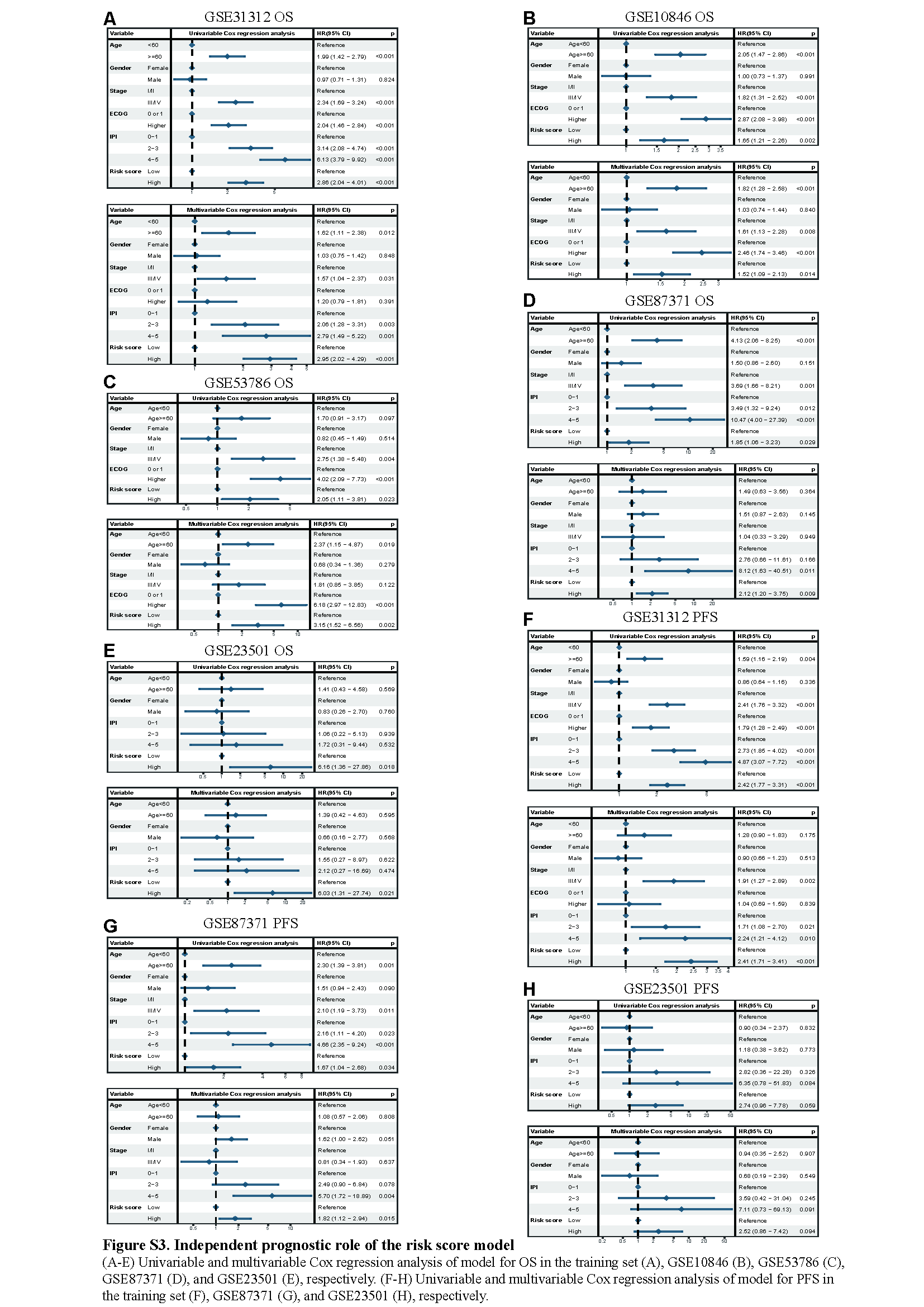

Supplement: Supplementary file 4 [file Image3.TIF]

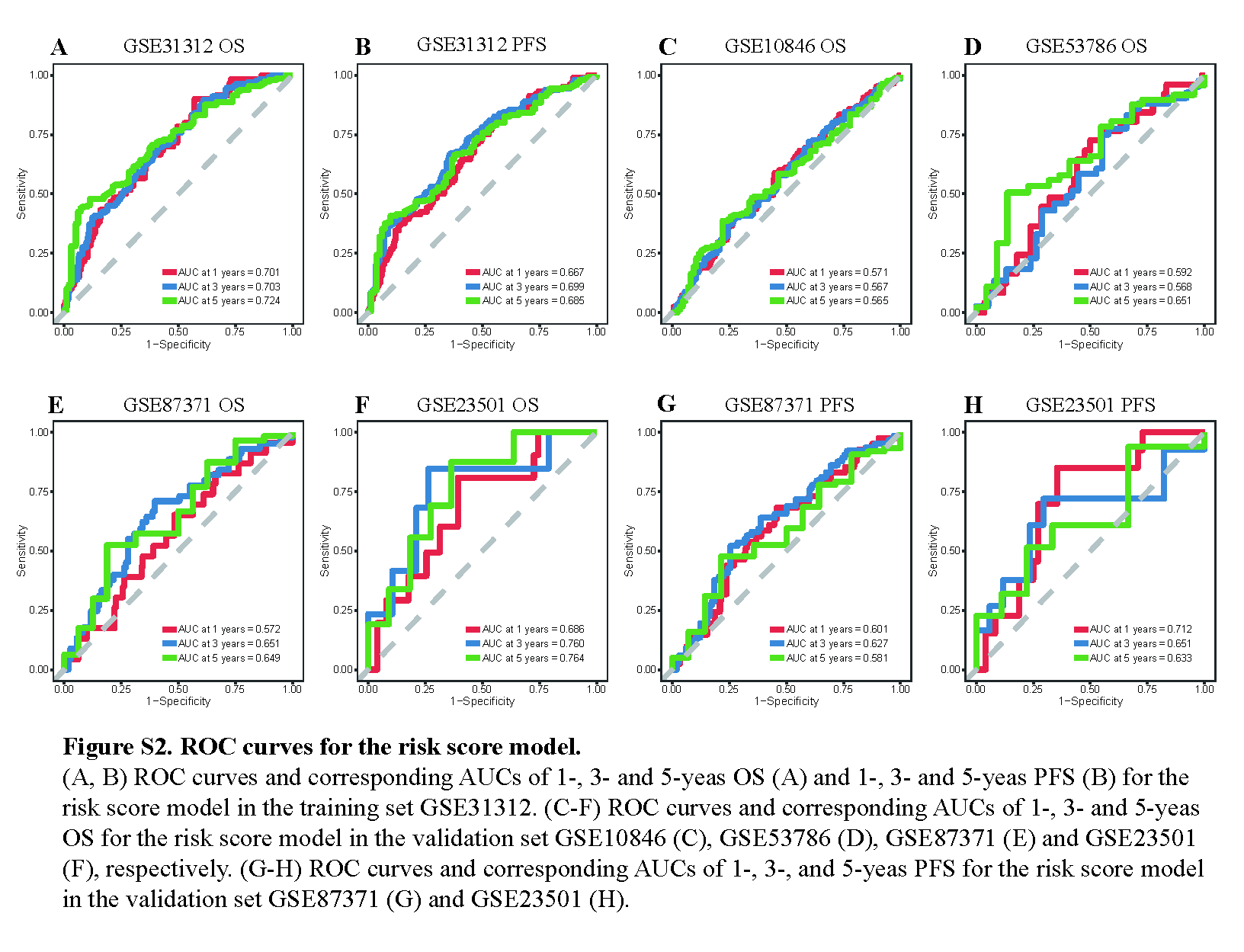

Supplement: Supplementary file 5 [file Image2.TIF]

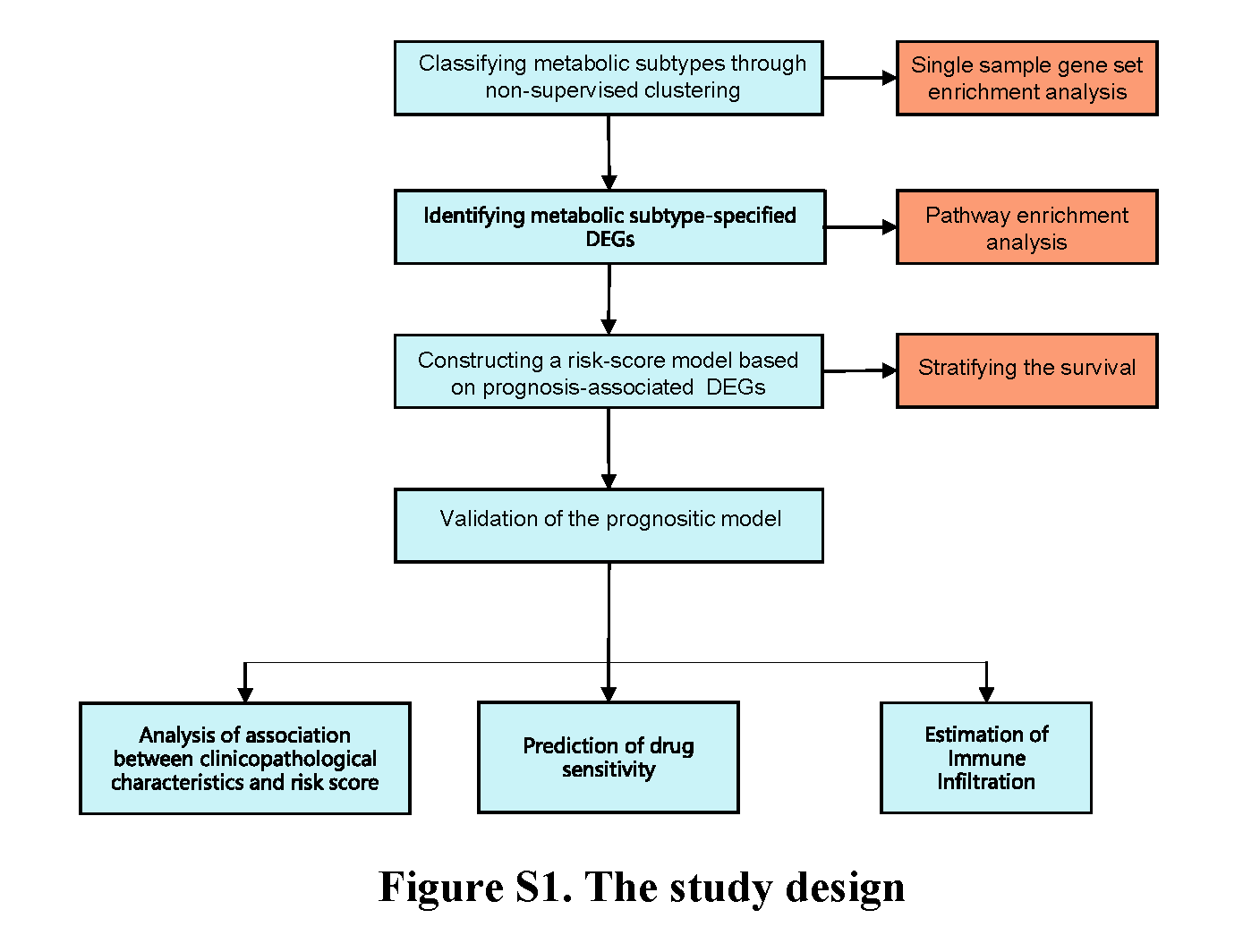

Supplement: Supplementary file 6 [file Image1.TIF]

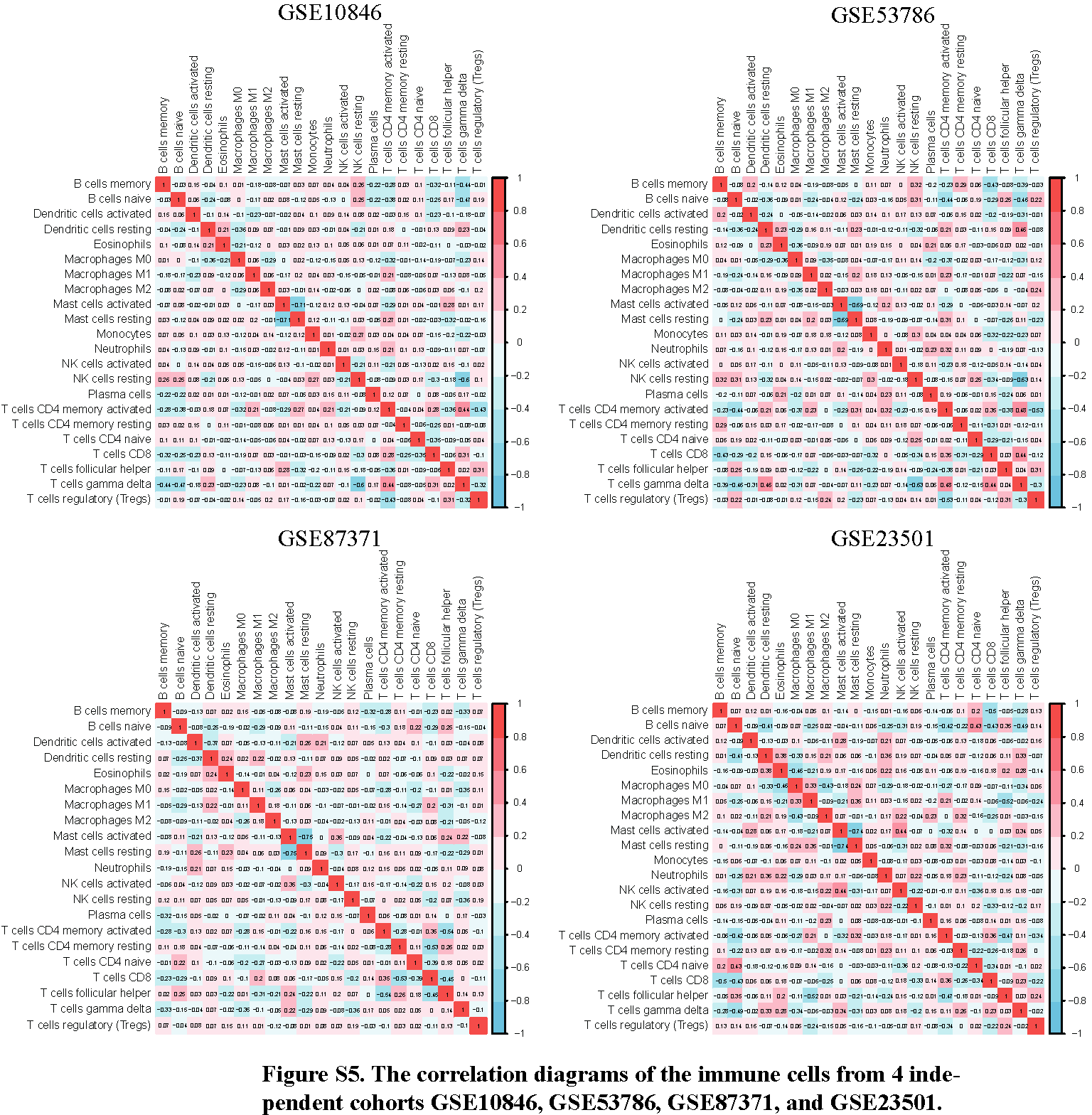

Supplement: Supplementary file 8 [file Image5.TIF]

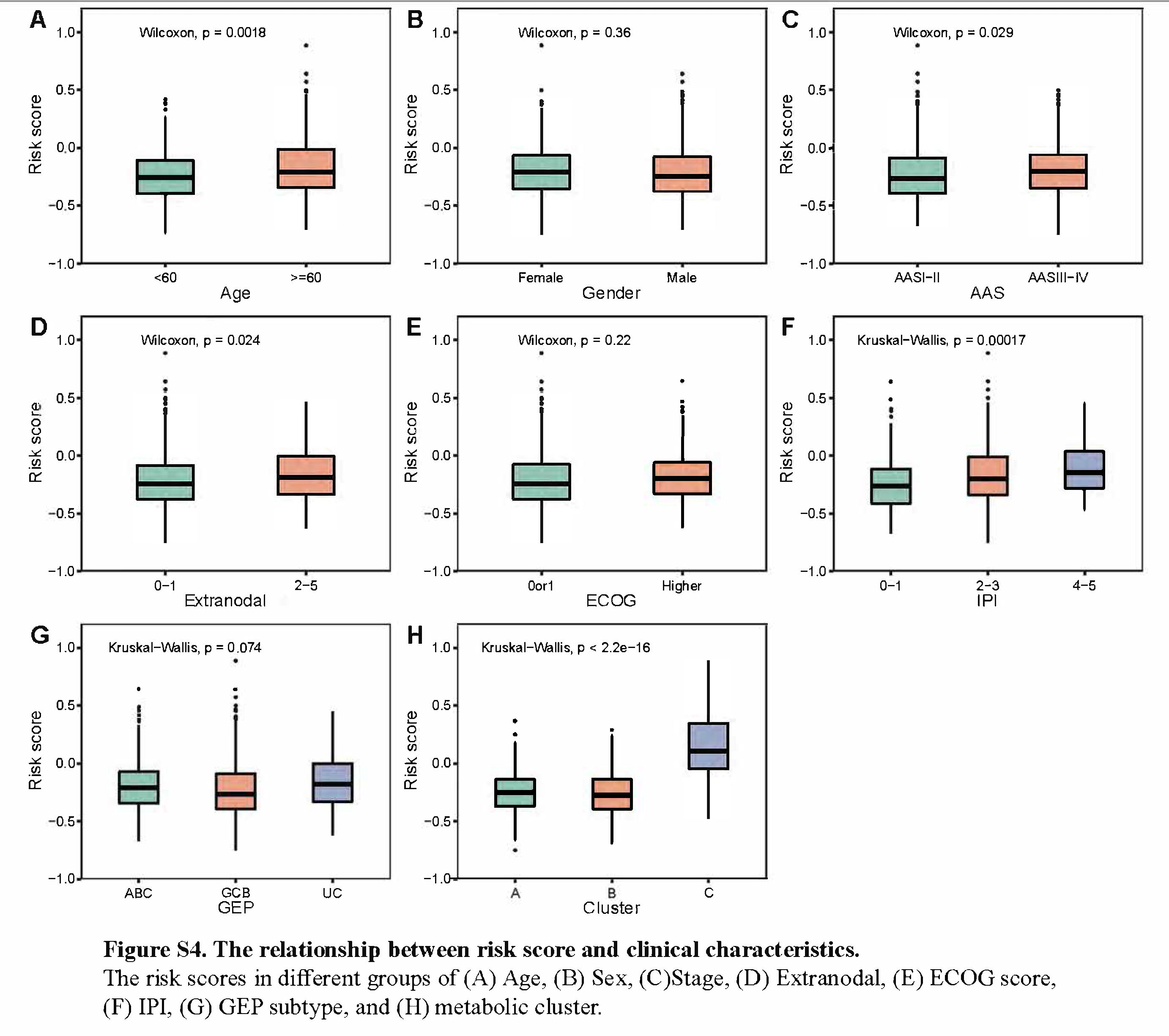

Supplement: Supplementary file 10 [file Image4.TIFF]
